# A rare case of asymptomatic left lung agenesis in an adult woman

**DOI:** 10.5339/qmj.2024.qitc.26

**Published:** 2024-04-08

**Authors:** Aasir M. Suliman, Theeb O. Sulaiman, Mona Allangawi

**Affiliations:** 1 Pulmonology Department, Hamad General Hospital, Hamad Medical Corporation, Doha, Qatar Email: asuliman4@hamad.qa

**Keywords:** Lung agenesis, lung aplasia, congenital lung anomaly

## Background

Pulmonary agenesis is a rare congenital anomaly characterized by the complete absence of pulmonary vessels, bronchi, and parenchyma. It can be unilateral or bilateral.^1^ It usually presents in the neonatal period or early childhood with various clinical presentations ranging from asymptomatic to recurrent episodes of wheezing or pneumonia and severe respiratory distress leading to chronic respiratory failure.^2^ Presentation in late adulthood is rare. Here we present a case of left pulmonary agenesis that was diagnosed incidentally in the fourth decade of life.

## Case Presentation

**Figure 1.**
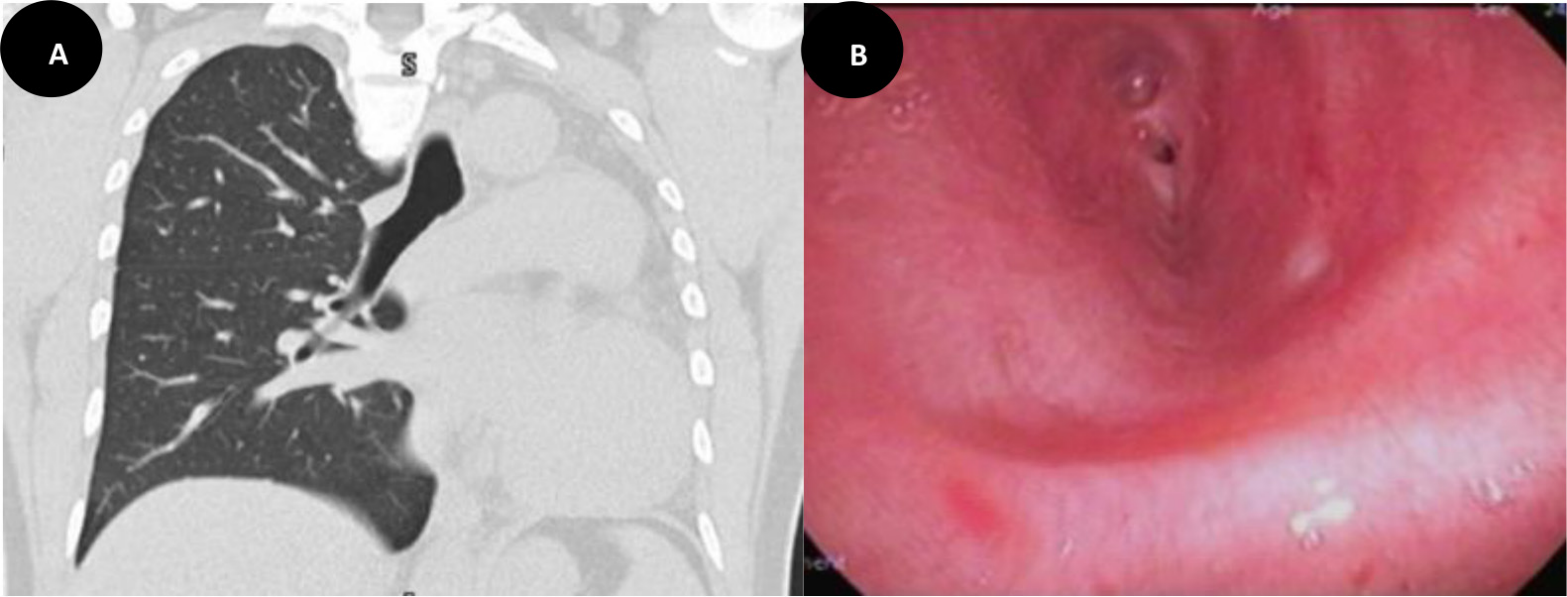
A coronal CT chest view shows a complete absence of left lung with a left side mediastinum shift and hyperinflation of the right lung (A). A bronchoscopy view demonstrates a rudimentary left main bronchus with complete absence of the remaining left bronchial tree (B).

A 38-year-old healthy woman with an unremarkable medical and surgical history was referred to our institution due to an abnormal chest X-ray during routine immigration screening. The X-ray revealed complete opacification of the left lung with an ipsilateral mediastinal shift. A subsequent chest computed tomography (CT) confirmed the absence of the left lung with hyperinflation of the right lung, indicating left lung agenesis (Figure 1A). A confirmatory flexible bronchoscopy revealed a rudimentary left main bronchus with complete absence of lung tissue (Figure 1B).

## Conclusion

Presentation of unilateral lung agenesis in late adulthood, as seen in the present case, is rare. Although it is uncommon, it is important to consider it in the differential diagnosis with the other pathologies that present with an opaque hemithorax and an ipsilateral mediastinal shift on radiography, such as lung collapse, pneumonectomy, pulmonary destruction, pleural effusion, lung cancer, and diaphragmatic hernia. Asymptomatic patients with no other congenital abnormalities do not require treatment. However, early diagnosis may prevent complications and unnecessary interventions, thereby improving survival.^3^

## Conflict of Interest

The authors declare that they have no known competing financial or personal interests.
